# Incentivizing Blood Donation: Systematic Review and Meta-Analysis to Test Titmuss’ Hypotheses

**DOI:** 10.1037/a0032740

**Published:** 2013-09

**Authors:** Claudia Niza, Burcu Tung, Theresa M. Marteau

**Affiliations:** 1 Department of Social Policy, London School of Economics, London, UK and Centre for the Study of Incentives in Health, King’s College London, London, UK; 2 Centre for the Study of Incentives in Health, King’s College London, London, UK; 3 Centre for the Study of Incentives in Health and Department of Psychology, King’s College London, London, UK

**Keywords:** blood donation, incentives, motivational crowding-out, behavioral economics, policy

## Abstract

***Objectives:*** Titmuss hypothesized that paying blood donors would reduce the quality of the blood donated and would be economically inefficient. We report here the first systematic review to test these hypotheses, reporting on both financial and nonfinancial incentives. ***Method:*** Studies deemed eligible for inclusion were peer-reviewed, experimental studies that presented data on the quantity (as a proxy for efficiency) and quality of blood donated in at least two groups: those donating blood when offered an incentive, and those donating blood with no offer of an incentive. The following were searched: MEDLINE, EMBASE and PsycINFO using OVID SP, CINAHL via EBSCO and CENTRAL, the Cochrane Library, Econlit via EBSCO, JSTOR Health and General Science Collection, and Google. ***Results:*** The initial search yielded 1100 abstracts, which resulted in 89 full papers being assessed for eligibility, of which seven studies, reported in six papers, met the inclusion criteria. The included studies involved 93,328 participants. Incentives had no impact on the likelihood of donation (OR = 1.22 CI 95% 0.91–1.63; *p* = .19). There was no difference between financial and nonfinancial incentives in the quantity of blood donated. Of the two studies that assessed quality of blood, one found no effect and the other found an adverse effect from the offer of a free cholesterol test (β = 0.011 *p* < .05). ***Conclusion:*** The limited evidence suggests that Titmuss’ hypothesis of the economic inefficiency of incentives is correct. There is insufficient evidence to assess their likely impact on the quality of the blood provided.

*The Gift Relationship* (1970) by Richard Titmuss is the seminal work against paying for blood. This work draws on the contrast between the U.S. blood supply system (mostly dependent on paid donors) and that in the U.K. (based entirely on unpaid donors), comparing the characteristics of blood donors, national statistics for blood supply and demand, and surveys of donors’ motivations. The book’s core premise is that altruistic blood donations are superior to a commercial provision of blood on the grounds of blood quality, economic efficiency, and moral value.

Titmuss’ prediction that payment would decrease blood quality was based on numerous reports by U.S. doctors of blood obtained from those with drug addictions and infectious diseases who successfully concealed their condition. In a market context, blood donors are motivated to withhold information about their health status as this disclosure may affect the price offered for their blood or even disqualify them as blood donors.

Contrary to common belief (e.g., Chmielewski, Bove, Lei, Neville, & Nagpal, 2012; Mellström & Johannesson, 2008), Titmuss did not predict that blood quantity would decrease if incentives were introduced. He did, however, consider the economic efficiency of paying for blood and the cost per unit of blood, which he claimed was higher in countries that paid donors because of a higher waste of blood and administrative costs. Blood quantity can be taken as a proxy for efficiency because, given the same amount of blood, the cost per unit from a paid source is higher than that from an unpaid source. In our review we will take the likelihood of donation as a proxy for economic efficiency of incentivized donations.

These arguments raised a heated discussion, particularly among economists, with criticism of Titmuss for his narrow view of market forces and lack of empirical support (Arrow, 1972; Solow, 1971). Proponents of the free-market for blood supply formulated the hypothesis that paying donors for blood would increase supply, based on earlier analyses (Cooper & Culyer, 1968).

But most importantly for Titmuss—and the most well-known feature of his work—is his defense of the superior moral value of altruistic blood donations compared with paid donations. He argued that decreasing the opportunity for altruistic donations with the offer of payment could have unpredictable negative consequences by limiting people’s freedom to give out of regard for the needs of others. Although this assumption was not based on empirical evidence, he presented survey data suggesting the negative impact of incentives in the former Soviet Union showing that after incentives were introduced, only 72% of donors reported they would keep donating if payments were withdrawn and only 50% of donors would donate as often as they currently did.

Despite the paucity of evidence, Titmuss’ manifesto for altruistic blood donation marked the start of discussions about perverse effects of incentivizing behavior that became known as *motivational crowding-out* (Promberger & Marteau, this issue). Motivational crowding-out is the umbrella term used in economics for the reverse of the relative price effect in economic theory, that is, when higher incentives lead to lower (not higher) supply (Bénabou & Tirole, 2006; Frey & Oberholzer-Gee, 1997; Kreps, 1997). Intrinsic motivation can be negatively affected when an external reward is offered, for example by changing the way the situation is perceived or by changing the individual’s self-perception as being controlled by the reward (Frey & Jegen, 2001). Evidence for motivational crowding-out in economics became the reduced supply once incentives are introduced.

There is also a large literature in psychology about the undermining effect of rewards on intrinsic motivation, developed shortly after Titmuss (Deci, 1975). This tradition, however, analyzes motivational crowding-out once incentives are removed. To our knowledge, no studies on blood donation provide data on the likelihood of donating after incentives are withdrawn. We therefore assessed motivational crowding-out in this review as defined in economic theory by considering changes in the blood supplied in the presence of incentives.

Titmuss’ work has influenced blood acquisition policies on a global level. The World Health Assembly (WHA) passed the resolution WHA 28.72 in 1975 urging member states to develop blood systems based on voluntary non-remunerated donation of blood. In 1997, the World Health Organization (WHO) recommended that all blood donations should come from unpaid voluntary donors. However, by 2006, only 49 of 124 countries surveyed had established this as a standard. The WHO reiterated their position in 2009 with the Melbourne Declaration on 100% Voluntary Non–remunerated Donation of Blood and Blood Components with the statement that “paid donation can compromise the establishment of sustainable blood collection from voluntary non-remunerated blood donors” (World Health Organization, 2009, p. 2).

Policies do, however, still differ across jurisdictions and regions. In the United States, blood is donated through various organizations registered with the U.S. Blood Bank, including blood centers, the Red Cross and hospitals, with some providing financial incentives to encourage donation (Domen, 1995). Financial incentives are most often used in the context of plasma donation, with concerns about the expected poorer quality of incentivized donations being abated by the use of technologies that can destroy viruses during the process of separating the blood from its plasma. In the U.K., the blood supply is managed through NHS Blood and Transplant, with blood only taken from unpaid voluntary donors, although there is a donor award scheme that introduces various gifts with respect to the amount of blood being donated. Paid donors remain major blood suppliers in some European countries, including Germany (Kretschmer, Weippert-Kretschmer, Slonka, Karger, & Zeiler, 2004).

The widespread idea that paying for blood could have unpredictable negative consequences raises the question of what constitutes payment and where a distinction may lie between financial incentives, such as cash or lotteries, and nonfinancial incentives, such as t-shirts, mugs, and medical tests. The former tends to raise more opposition, whereas the latter is more commonly accepted as a legitimate way to incentivize blood donation. Two observational studies claim that these nonfinancial incentives can be more effective than monetary payments to increase blood donation. In a survey of 467 blood donors in an Italian town, Lacetera and Macis (2010) found that donors reported they would stop being donors if given 10 Euros in cash, but not if a voucher of the same nominal value was offered instead. Costa-i-Font and colleagues (Costa-i-Font, Jofre-Bonet, & Yen, 2011) analyzed attitudes toward payment for blood in large representative samples of 15 European countries and concluded that those in favor of paid donations were less likely to have donated blood, whereas those favoring nonmonetary rewards were equally or more likely to be donors. In our work, we will include both types of incentives to test this claim.

Two reviews have considered the impact of incentives on the likelihood of giving blood (Godin, Vézina-Im, Bélanger-Gravel, & Amireault, 2012; Goette, Stutzer, & Frey, 2010). One is an unsystematic, narrative review of the literature that includes both observational and experimental studies (Goette et al., 2010). The authors conclude that incentives work relatively well in increasing blood supply. The other review was more systematic and focused on experimental studies of a range of interventions of which incentives were just one (Godin et al., 2012). Only two studies on incentives were included in this review (Ferrari, Barone, Jason, & Rose, 1985; Jason, Jackson, & Obradovic, 1986). From these two studies, the authors concluded it was not possible to make any claim about the impact of incentives.

Regarding blood quality, there are two reviews strictly based on observational studies that reported a higher prevalence of transfusion-transmissible viruses in blood acquired from paid donors (Eastlund, 1998; van Der Poel, Seifried, & Schaasberg, 2002). In keeping with these findings, a more recent study from Lithuania assessing both regular and first-time whole blood donors found that blood from first-time paid donors was of poorer quality (Kalibatas, 2008).

Our review aims to provide the first formal synthesis of evidence to assess the impact of offering financial incentives upon the quantity and quality of blood obtained.

## Method

We used the Cochrane Review handbook to guide the methods used in this review (Higgins & Green, 2011).

### Inclusion and Exclusion Criteria

The inclusion criteria were (a) published randomized studies in which participants in one group were offered an incentive for blood donation and in another group were not, and (b) that reported data on one or both of two outcomes: the proportion of people providing blood, and the quality of the blood provided. Incentives were defined as a good or service with a monetary value offered in exchange for blood. These could be described as compensation for resources spent in donation (most usually time) or as an explicit motivator. We excluded exchanges of little or no monetary value such as certificates or badges.

### Data Sources and Searches

We searched MEDLINE (1950 to December 2011), EMBASE (1980 to December 2011), and PsycINFO (1985 to December 2011) using OVID SP, and CINAHL (1982 to December 2011) via EBSCO. The search strategies used both keywords and medical subject headings. We searched for relevant systematic reviews in the Cochrane Central Register of Controlled Trials (CENTRAL, the Cochrane Library, December 2011) as well as the OVID SP databases (1985 to December 2011), EconLit via EBSCO (1996 to December 2011), JSTOR Health and General Science Collection (1886 to December 2011 in Economics), and Google, using terms related to incentives and blood donation.

**Figure 1 fig1:**
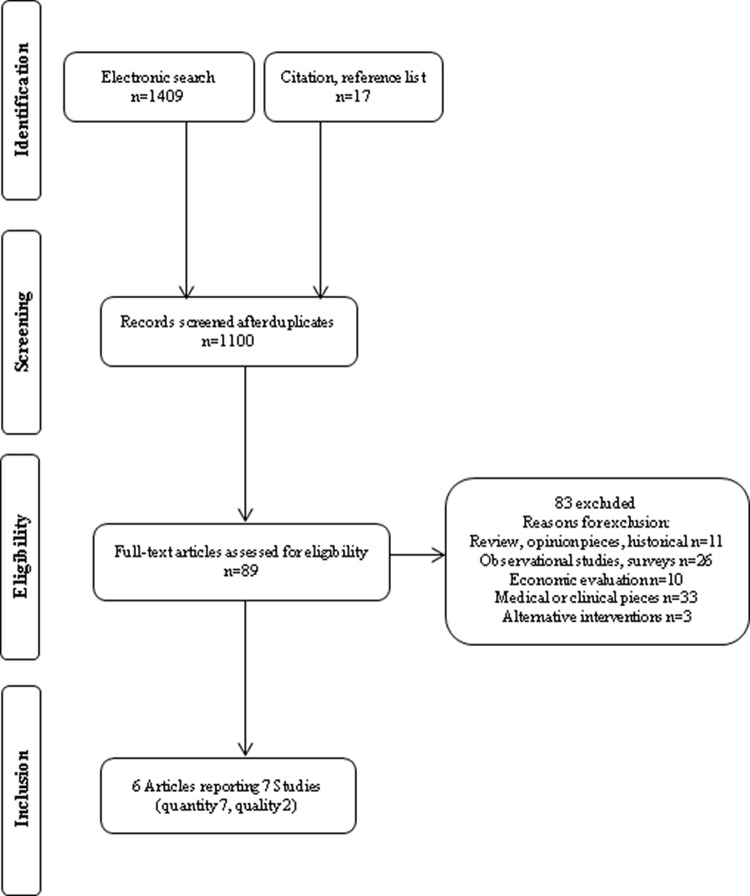
Study selection.

The initial search yielded 1100 abstracts (see Appendix A), which resulted in 89 full papers being assessed for eligibility. The large number of papers excluded at the screening stage was mostly a result of the retrieval of clinical trials that assessed quality of blood retrieved but not following the offer of incentives. Six papers, reporting seven studies between them, met the eligibility criteria for our review (see Figure 1). Of these, two studies did not report data in a form that could be extracted for meta-analysis (Goette & Stutzer, 2008; Goette, Stutzer, Yavuzcan, & Frey, 2009 Study 2). Requests to the authors for the data in an extractable form were unsuccessful.[Fig-anchor fig1]

### Data Extraction and Quality Assessment

Two review authors prescreened all search results (titles and abstracts) against the selection criteria for possible inclusion, and those selected by both review authors were subjected to a full-text assessment. Two review authors independently assessed the selected full-text articles for inclusion, resolving any discrepancies by consensus. Variables of interest included study participants, study design, incentive, outcome measure, and results.

Risk of bias was assessed by two authors in accordance with the guidelines of the Cochrane Consumers and Communication Review Group (Higgins & Green, 2011), which recommends the explicit reporting of individual elements that affect risk of bias, including:

1 Sequence generation: classified as adequate if carried out using true randomization and not quasi-randomization, such as by day of week, date of birth, or sequence;
2 Allocation concealment: classified as adequate if allocation is concealed from the purveyor of risk information, researchers, and the participant at least until the point of allocation to groups;
3 Blinding: classified as adequate if participants, personnel, and outcome assessors are blind to allocation.
4 Incomplete outcome data: classified as adequate if attrition data are clearly reported and there is no evidence of differential drop out in the intervention and control groups;
5 Selective outcome reporting: classified as adequate if data are provided for all outcomes specified in the study protocol, or where this may be unavailable, in the methods section;
6 Other sources of bias, including baseline comparability: classified as adequate if groups are comparable at baseline or any differences at baseline are adjusted for in the primary analysis; and validation of measures, classified as adequate if there is evidence of reliability and validity reported in the study or published elsewhere.

### Data Synthesis and Analysis

Three studies that were presented in two papers reported outcomes from more than one incentive condition (Lacetera, Macis, & Slonim, 2012; Mellström & Johannesson, 2008). Because these observations are not independent, the results from only one incentive condition per study are included in the meta-analysis. For Mellström and Johannesson (2008) we include the results from the offer of the fixed incentive (and not the offer of a choice between an incentive and a donation to charity), thus making the intervention more comparable with other interventions in the review. In Lacetera et al. (2012), three sizes of incentive were offered ($5, $10, $15), and we performed separate analyses to independently include each incentive size.

The effect size is reported using odds ratios (OR), with an OR greater than one favoring the intervention group. We obtained pooled effect sizes with 95% confidence intervals using a random effects model.

## Results

Of the seven included studies (see Table 1), three were conducted in the United States, three in Switzerland, and one in Sweden. Four involved previous donors, two involved previous non-donors, and one comprised both types of donors. The financial incentives that were offered included money, lottery tickets, and cholesterol tests, with an estimated value of between $3 and $15 each.[Table-anchor tbl1]

The seven studies involved 93,328 participants with an age range from under 20 to 65. The gender mix among participants ranged from 39% to 60% women.

### Quantity of Blood Provided

The seven included studies assessed the impact on the likelihood of donating following the offer of a financial incentive. However, it was only possible to pool data from five of the seven studies. In Lacetera et al. (2012) the authors compared four conditions: (a) advertised reward and informed, (b) advertised and uninformed, (c) surprise reward, and (d) no reward. We could not access the raw data for the control group, so the comparison presented is between (c) surprise reward and (a) advertised and informed reward. The former acted as a control because, although there was the offer of an incentive, participants were unaware of this and therefore their behavior cannot be attributed to the incentive. The raw data are not presented in the paper but were kindly provided to us by the authors.

**Figure 2 fig2:**
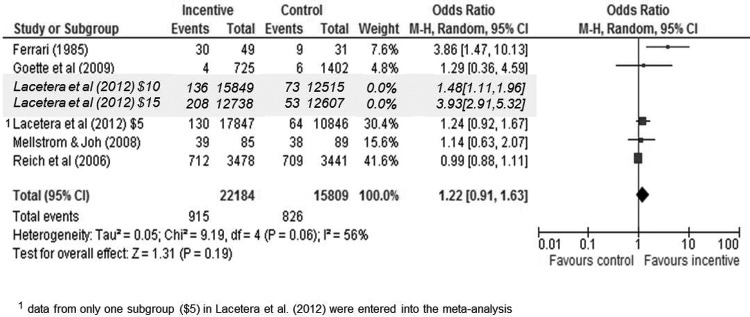
Impact of financial incentives upon likelihood of providing blood.

In the five studies included in the meta-analysis (see Figure 2), the likelihood of blood donation was similar when financial incentives were offered and when they were not (OR = 1.22 95% CI 0.91–1.63 *p* = .19). This estimate was calculated using the data from Lacetera et al. (2012) for the $5 incentive but it holds for the other two higher incentive sizes: $10 (OR = 1.33 95% CI 0.94–1.89 *p* = .11) and $15 (OR = 1.85 95% CI 0.83–4.11 *p* = .13). There was no significant evidence of between-study heterogeneity with χ^2^ = 9.19 *p* = .056 and no sign of small-study effects with Egger’s test = 1.51 *p* = .132. Begg’s test was not significant (z = .98 *p* = .327), suggesting an absence of publication bias. Study estimates show no pattern by publication year.[Fig-anchor fig2]

In both the studies that could not be included in the meta-analysis, individuals in the experimental groups were offered a free cholesterol test as an incentive, which had no impact on the likelihood of providing blood in either study (Goette & Stutzer, 2008; Goette et al., 2009 Study 2). In one study (Goette et al., 2009) a lottery was offered to a second experimental group. This had no main effect but among donors with a previous low rate of donation, it increased the likelihood of donation (by an estimated 9%), with no impact on those with previously high rates of donation.

### Subgroup Analyses

Based on very small subgroup analyses, none of the incentive types seemed to be effective: cash OR = 1.14, *p* = .67, 95% CI 0.63–2.07 (*n* = 1), vouchers OR = 2.13, *p* = .11, 95% CI 0.85–5.31 (*n* = 2), and gifts OR = 0.99, *p* = .92, 95% CI 0.89–1.12 (*n* = 2). Further analyses similarly showed no difference when participants were previous non-donors (OR = 1.16, *p* = .58) or when they were previous donors (OR = 1.06, *p* = .57).

### Quality of Material Provided

Two studies assessed the impact of offering financial incentives to existing donors on the quality of blood provided as indicated by the rejection rate (Goette & Stutzer, 2008; Lacetera et al., 2012). Lacetera et al. (2012) reported that incentives (gift cards) did not change the proportions of rejected donations, which were 0.12% and 0.14% in the advertised and non-advertised sites, respectively. Goette and Stutzer (2008) found no effect on quality of donations following the offer of a lottery ticket but the offer of a cholesterol test increased the proportion of donations rejected (β = 0.011, *p* < .05).

### Assessment of Risk of Bias

Table 2 presents the assessment of risk of bias for included studies. The pattern of findings suggests a moderate risk of bias from a failure in any study to specify methods of randomization, and those assessing outcomes not being blind to group allocation. All studies had adequate presentation of outcome data (despite two being excluded from meta-analysis because they did not present outcome data in ways that allowed them to be used), and there was no evidence of selective reporting or other noticeable sources of bias.[Table-anchor tbl2]

## Discussion

From the few studies that met the eligibility criteria, we found no impact of offering financial incentives on the quantity of blood given. With respect to blood quality, only two studies met the inclusion criteria. One study reported no impact of a gift card on the quality of blood provided. The other study reported poorer quality donations when the incentive offered was a medical test but not when the incentive was a lottery ticket. There was no evidence of motivational crowding-out if operationalized as a lower blood supply when incentives are offered.

The strength of this review is that it is the first to our knowledge that attempts to examine the evidence for Titmuss’ influential hypotheses concerning the adverse effects of using incentives to encourage blood donation. We have revealed the paucity of experimental evidence, as well as different conclusions to earlier, unsystematic reviews. In contrast to the unsystematic narrative review by Goette et al. (2010), based on a mixture of observational and experimental studies, the results of our more robust review do not corroborate their conclusions that incentives increase blood donation. Using more robust search methods compared with Godin et al. (2012), our review is based on a larger number of studies, allowing us to draw some conclusions.

The studies were heterogeneous both in terms of the interventions and the populations studied. The incentives offered included t-shirts, cholesterol tests, money, gift cards, and lottery tickets, and their value varied from $3 to $15, studied in populations from different countries. Nevertheless, there was no evidence of statistical between-study heterogeneity, which suggests that the overall estimate was robust.

The small number of included studies limited the meaningfulness of subgroup analyses to explore the impact of several potential effect modifiers. Previous blood donation could be an important moderator of incentive effects although our analyses showed no differences between first-time and previous donors. Two surveys (Costa-i-Font et al., 2011; Lacetera & Macis, 2010) claimed that nonmonetary incentives would be more effective than monetary payments to increase blood donation. Our results showed that none of the types was effective, although the pattern of findings suggested that vouchers may be more effective. Mellström and Johannesson (2008) identified a post hoc gender effect (significant crowding-out effect in women and no effect on men), but this result was not replicated in other studies with more power.

It could be argued that the size of the incentives offered was not sufficient to motivate behavior. However, there is considerable evidence that very small incentives can change behavior in other health domains including disease screening and medical adherence (e.g.,Helmus, Saules, Schoener, & Roll, 2003; Malotte, Rhodes, & Mais, 1998; Tulsky et al., 2000; Volpp et al., 2008), suggesting that blood donation may be more resistant to the impact of incentives.

Some methodological limitations and their consequent risk of bias in the included studies should to be taken into account when drawing any conclusions from this review. The assessment of risk of bias suggested a moderate risk from lack of detail both in the methods of randomization, and blindness of outcome assessors to group allocation. All but two of the studies were powered to detect small effects of incentives, with the exception of Ferrari et al. (1985) and Mellström and Johannesson (2008).

### Implications for Practice and Research

We found support for Titmuss’ hypothesis that incentives are economically inefficient: offering incentives did not increase the quantity of the blood donated and introduced additional costs. The fact that incentives had no impact on the quantity of blood, as would be predicted by the price effect in classical economic theory, is one reason against its use in practice. Titmuss’ concerns about blood quality were mostly related to previous non-donors and how the offer of incentives could decrease blood quality by attracting more at-risk donors. The two studies that reported on blood quality both involved previous donors, thus providing at best a partial test of this hypothesis.

The empirical tests of Titmuss’ hypotheses, however, are not the only considerations. Moral judgments that are not about the readily measurable consequences also play a part. Titmuss’ arguments against the use of incentives in blood donation had deep roots in his humanist conception of social policy as a tool for human progress that should be protected from what he considered to be exploitative economic interests. He endorsed blood donation as a core example of how society should be governed by relationships characterized by reciprocity. Regardless of the effectiveness of incentives to increase blood stocks, paid donors become blood sellers and blood donation becomes blood supply. Two recent analyses of the role of money markets for human blood (Healy, 2006) and beyond (Sandel, 2012) share this perspective, reflecting “protected values” (Baron & Spranca, 1997) or “sacred values” (Tetlock, Kristel, Elson, Green, & Lerner, 2000), echoed in the widely held view that some human exchanges should not be traded off against money. The insight provided by the few studies included in this review is limited not only in quantity but also by quality, as reflected in study designs. Uncertainty would be reduced by additional experimental studies in which the size and type of incentives were varied, in first-time and previous donors.

We found no support for motivational crowding-out, operationalized as a decrease in blood supply in the presence of incentives. It remains unknown, however, whether incentives had some crowding out effect on who donated rather than how many donated. For example, incentives may have distanced voluntary donors and attracted new, incentive-driven donors without affecting the overall number of donors. The longer-term outcomes of incentivizing need also to be studied. We found no studies that assessed the impact of incentivizing on subsequent likelihood of donating when incentives were not offered.

The extent to which incentives that are larger than those included in this review would effectively increase blood donation remains unknown. Also unknown is the cost-effectiveness of larger incentives.

In conclusion, the limited evidence suggests that Titmuss’ hypothesis of the economic inefficiency of incentives may be correct. There is insufficient evidence to assess their likely impact on the quality of the blood provided.

## Figures and Tables

**Table 1 tbl1:** Characteristics of Included Studies

Authors	Country	Participants and setting	Type of donor	Groups	
				Control group	Incentive group
Ferrari et al. (1985)	US	Posters and bulletins posted around a US University campus with announcement about time and place of blood donation	Both first-time and previous donors	*n* = 31Peer altruism. Students were informed that their peers would be donating blood	*n* = 49Offer of coupons redeemable at local merchants for free or reduced-price merchandise and a raffle (tickets to Broadway play, college football game)
Reich et al. (2006)	US	Blood Centers in San Francisco and Arizona; Outcome second and third donations of first-time donors within 6 months	Previous donors	*n* = 3,441	*n* = 3,478Offer of a t-shirt
Mellström & Johannesson (2008)	Sweden	Regional Blood Centre Gothenburg Sweden; Primary outcome health check for blood donation	First-time donors	*n* = 89	*n* = 85 Offer of $7 *n* = 88 Choice between $7 and donation to charity
Goette & Stutzer (2008)	Switzerland	Zurich Blood Donation Service of the Swiss Red Cross; individuals registered in the database invited to donate blood again	Previous donors	*n* = 2,950	*n* = 4,431 Offer of a free cholesterol test *n* = 1205 Offer of a lottery ticket from Swiss State Lottery
Goette et al. (2009) Study 1	Switzerland	Zurich Blood Donation Service of the Swiss Red Cross	First-time donors	*n* = 725	*n* = 1,400 Offer of a free cholesterol test
Goette et al. (2009) Study 2	Switzerland	Zurich Blood Donation Service of the Swiss Red Cross 8269 previous donors	Previous donors	*n* = 1,968	*n* = 3,812 Offer of a free cholesterol test
Lacetera et al. (2012)	US	The American Red Cross (ARC) conducted 14,029 blood drives in US northern Ohio between May 2006 and October 2008	Previous donors	$5 *n* = 10,846 $10 *n* = 12,515 $15 *n* = 12,607 Total *n* = 35,968	$5 *n* = 17,847 $10 *n* = 15,849 $15 *n* = 12,738 Total *n* = 46,434 The different amounts refer to gift cards redeemable for food, gasoline and general merchandise.

**Table 2 tbl2:** Assessment of Risk of Bias

Study	Sequence generation	Allocation concealment	Blinding	Incomplete outcome data	Selective reporting	Other sources of bias
Ferrari et al. (1985)	Unclear – “10 female volunteers were instructed to randomly use one of two strategies” p. 792	Researchers aware of allocation to groups	Only participants blind to allocation	Adequate	Adequate	Adequate “no difference existed between (…) conditions in sex and donation history”
Reich et al. (2006)	Unclear – “each donor had an equal chance of being randomized into groups” p. 1091	Recruitment staff aware of allocation to groups	Only participants blind to allocation	Adequate	Adequate	No baseline comparison
Mellström & Johannesson (2008)	Unclear – “participants were randomly allocated into three groups” p. 848	Unclear	Unclear - participants blinded to allocation	Adequate	Adequate	Adequate
Goette & Stutzer (2008)	Quasi - cluster randomization per donation center and per day of week	Adequate. Allocation concealed to staff and participants	Adequate. Staff and participants blinded to allocation	Adequate	Adequate	Adequate control of baseline differences
Goette et al. (2009) Study 1	Unclear – “treatments were randomly assigned to mail orders” p. 527	Unclear	Participants blinded to allocation	Adequate	Adequate	Adequate control of baseline differences
Goette et al. (2009) Study 2	Unclear – “randomly invited” p. 527	Unclear	Participants blinded to allocation	Adequate	Adequate	Adequate control of baseline differences
Lacetera et al. (2012)	Unclear – “randomly selected” p. 17	Researchers aware of allocation, unclear for staff	Participants blinded to allocation	Adequate	Adequate	Adequate control of baseline differences

## A Search Strategy

Keywords (kw) and Subject Headings (su) Used in the Search

**Table:**

	MEDLINE	EMBASE	PsycInfo	CINAHL
Financial incentives	Monetary incentive	Incentives	Incentives (su)	Physician incentive plans (su)
	Financial incentive	Monetary incentive*	Monetary incentives (su)	Incentive*
	Reimbursement, incentive (su)	Financial incentive*	Financial incentive (kw)	Finance*
	Physician incentive plans (su)	Rewards (su)	Rewards (su)	Monetary*
	Incentive*	Incentive*	Monetary rewards (su)	Lottery
	Reward (su)	Cash	Rewards (kw)	Cash
	Monetary*	Monetary*	Cash (kw)	Reward
	Cash	Lottery	Monetary* (kw)	Reward (su)
	Lottery	Compensation (su)	Lottery (kw)	Compensation
	Compensa*	Compensation	Compensation (kw)	Pay*
	Pay*	Lottery	Pay*	Sell*
	Sell*	Pay*	Sell*	Remunera*
	Financ*	Sell*	Finance*	
	Remunera*	Remunera*	Remunera*	
Blood	Blood donors (su)	Blood don*	Blood don*	Blood for money
	Blood don*	Morey for blood	Blood trans*	Paid blood donation
	Paid blood*	Paid blood donation	Money for blood	Blood don*
	Money for blood	Blood donation	Paid blood donation	Blood donors (su)
	Blood tansfusion (su)	Blood donor su		Blood transufion (su)
				Exchange transfusion (su)
				Whole blood (su)
