# Correction to Niza, Tung, and Marteau (2013)

**DOI:** 10.1037/hea0000035

**Published:** 2013-10-14

The article “Incentivizing Blood Donation: Systematic Review and Meta-Analysis to Test Titmuss’ Hypotheses” by Claudia Niza, Burcu Tung, and Theresa M. Marteau (*Health Psychology*, Vol. 32, No. 9, pp. 941–949. doi: 10.1037/a0032740), was published with the incorrect copyright line. The copyright line should have read ©2013 The Author(s). Additionally, the Author Note should have stated, “This article has been published under the terms of the Creative Commons Attribution License (http://creativecommons.org/licenses/by/3.0/), which permits unrestricted use, distribution, and reproduction in any medium, provided the original author and source are credited. Copyright for this article is retained by the author(s). Author(s) grant(s) the American Psychological Association the exclusive right to publish the article and identify itself as the original publisher.” The online version of this article has been corrected.

